# Supplementary figures and images for: Emergence and Spread of a B.1.1.28-Derived P.6 Lineage with Q675H and Q677H Spike Mutations in Uruguay
Source: Viruses. 2021 Sep 10;13(9):1801. doi: 10.3390/v13091801 (PMC8473254; doi:10.3390/v13091801)

A

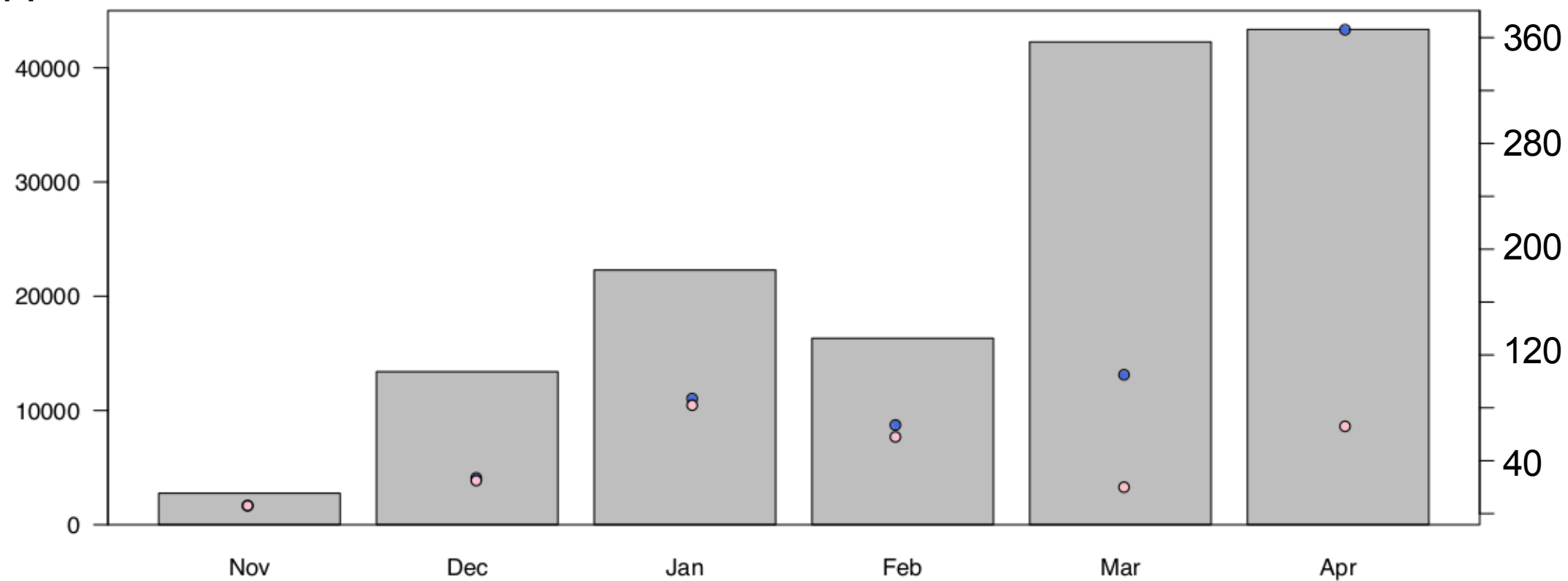

B

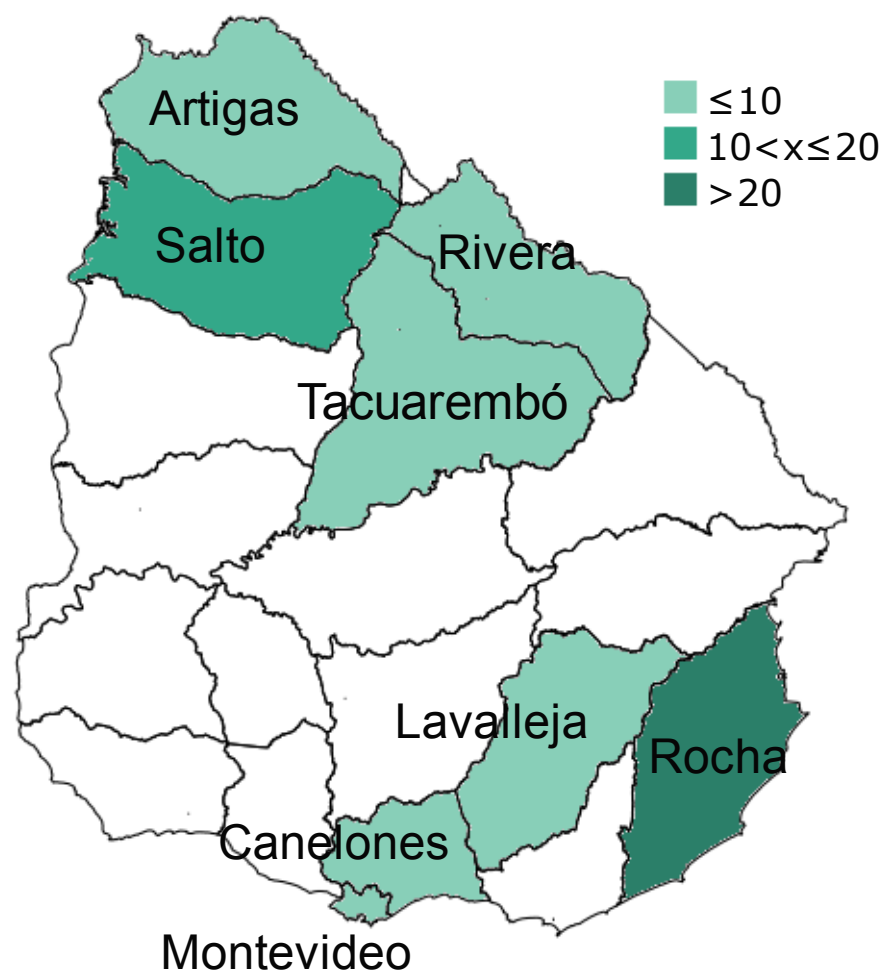

C

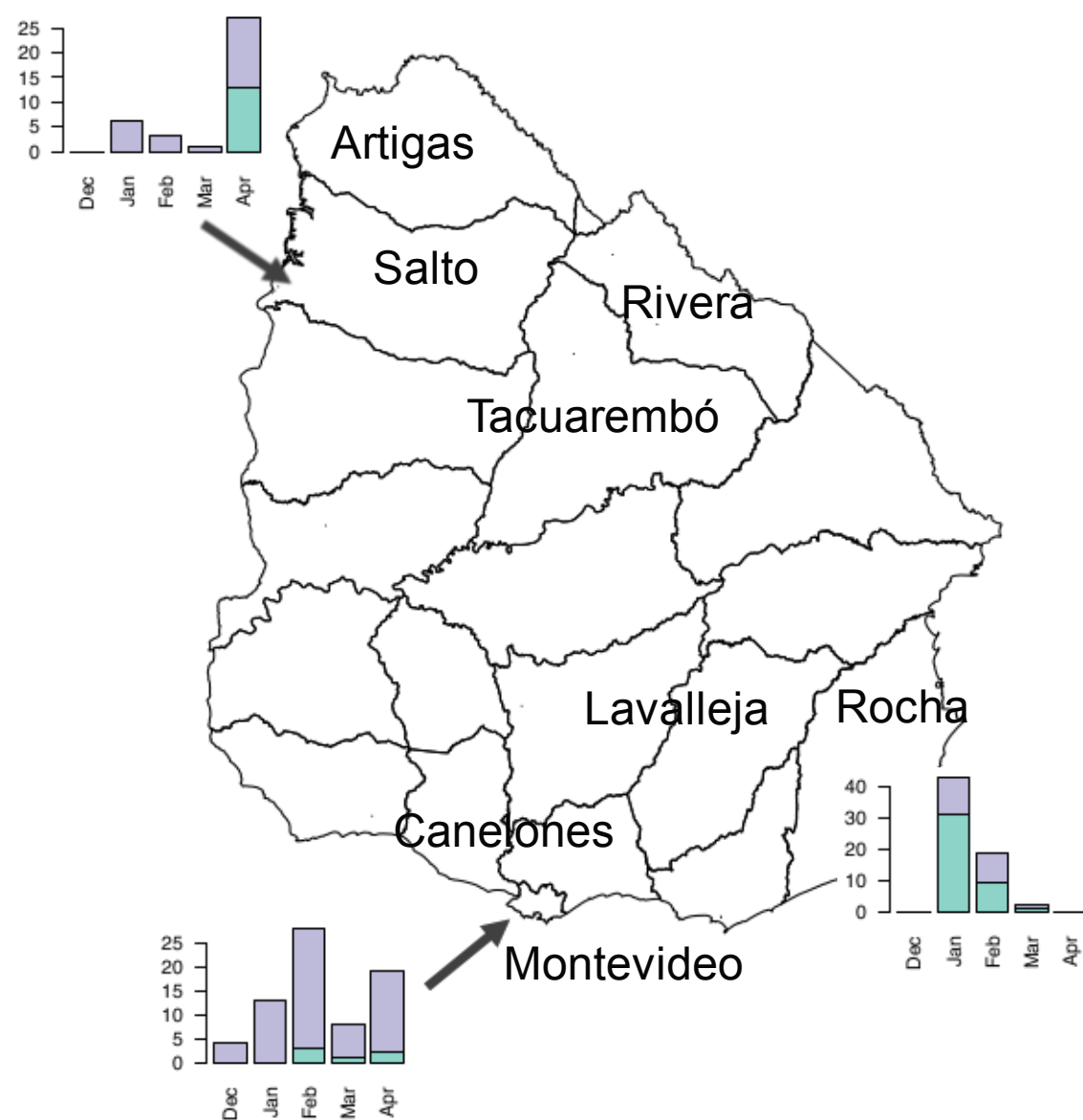

Supplement: Supplementary file 1 [file viruses-13-01801-s001.zip › sup_mat/FigureS1_Viruses.pdf]

location

- BR-Central-West
- BR-North
- BR-Northeast
- BR-South
- BR-Southeast
- Uruguay

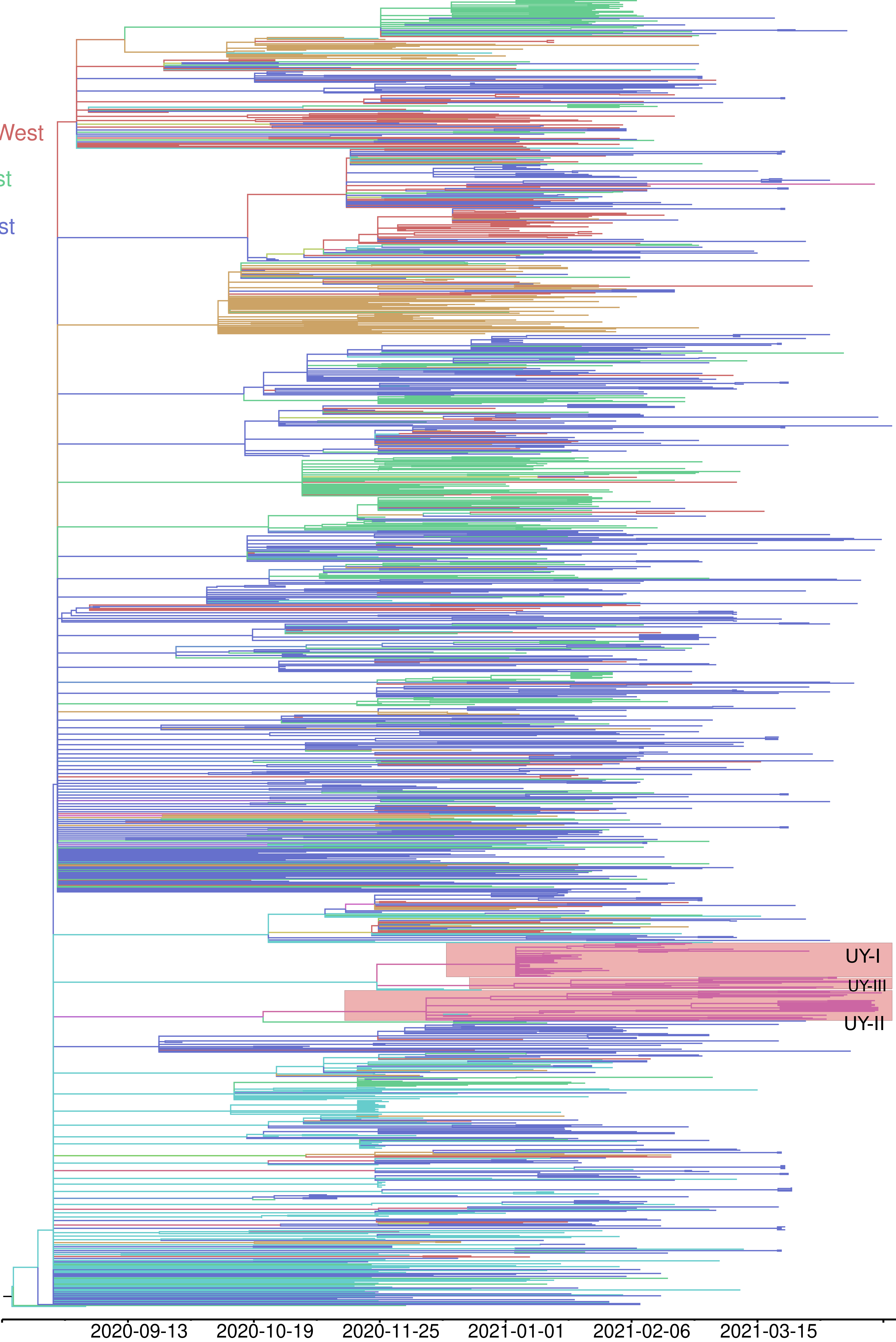

Supplement: Supplementary file 1 [file viruses-13-01801-s001.zip › sup_mat/FigureS4_pastml_p2.pdf]

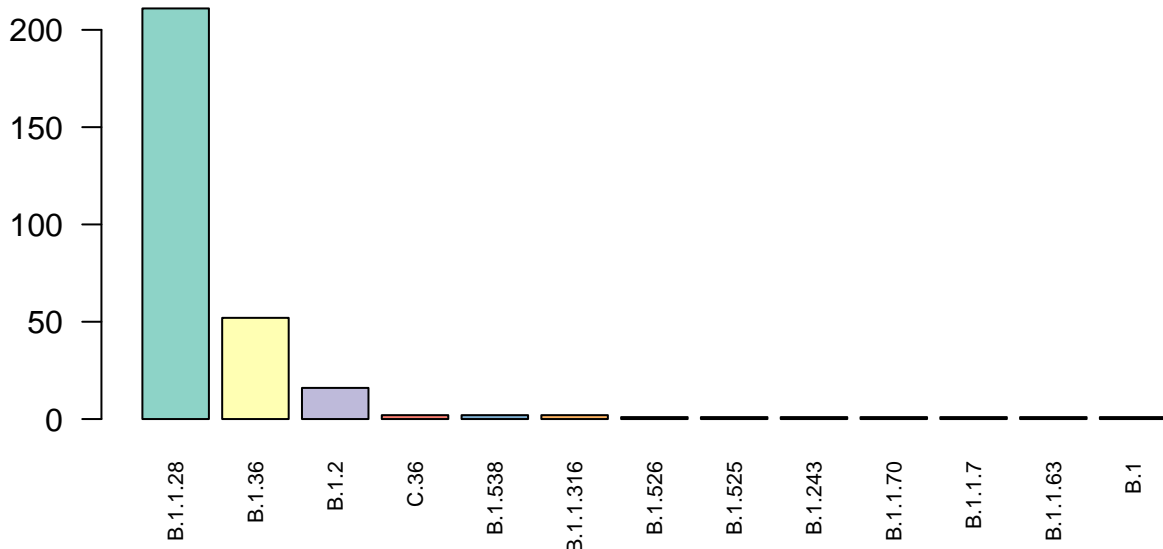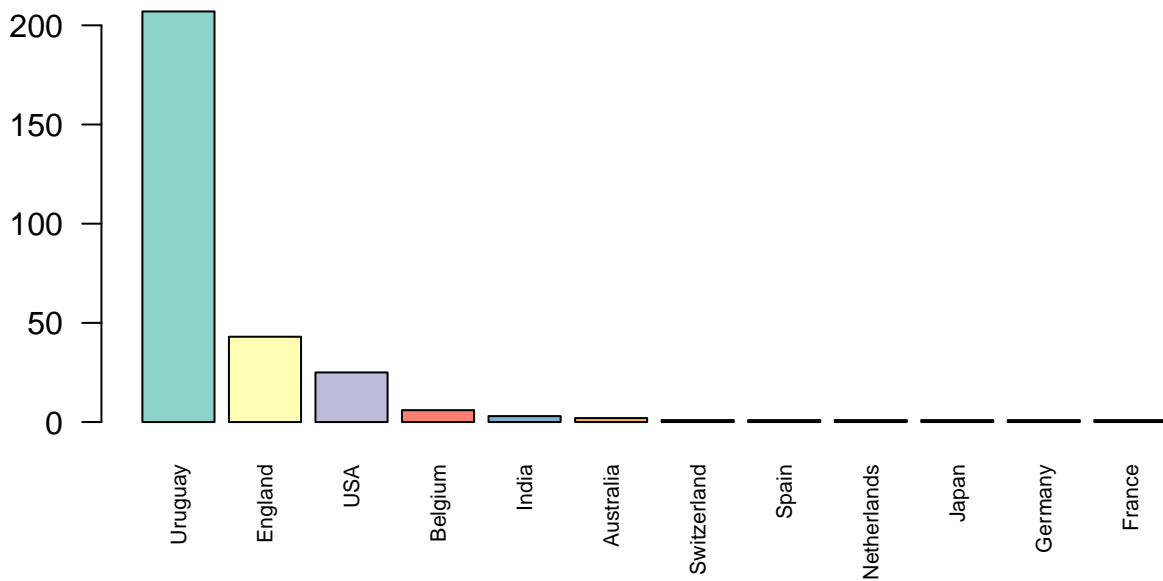

Supplement: Supplementary file 1 [file viruses-13-01801-s001.zip › sup_mat/FigureS5_numeros_b1128_paises_linajes.pdf]
