# Supplementary material for: Emergence and Spread of a B.1.1.28-Derived P.6 Lineage with Q675H and Q677H Spike Mutations in Uruguay
Source: Viruses. 2021 Sep 10;13(9):1801. doi: 10.3390/v13091801 (PMC8473254; doi:10.3390/v13091801)

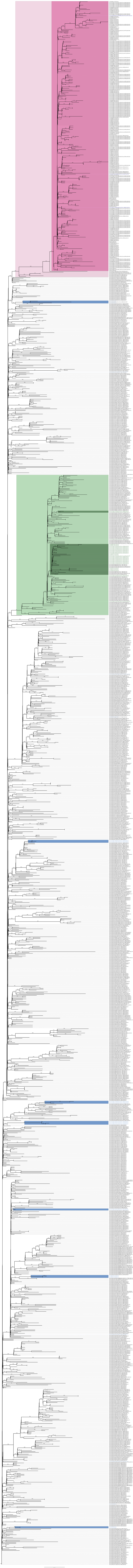

Supplement: Supplementary file 1 [file FigureS2_B1128-con-P7.tree.pdf › sup_mat/FigureS2_B1128-con-P7.tree.pdf]
