# Supplementary material for: Emergence and Spread of a B.1.1.28-Derived P.6 Lineage with Q675H and Q677H Spike Mutations in Uruguay
Source: Viruses. 2021 Sep 10;13(9):1801. doi: 10.3390/v13091801 (PMC8473254; doi:10.3390/v13091801)
Supplement: Supplementary file 1 [file FigureS3_pelotas_pastml.pdf › sup_mat/FigureS3_pelotas_pastml.pdf]

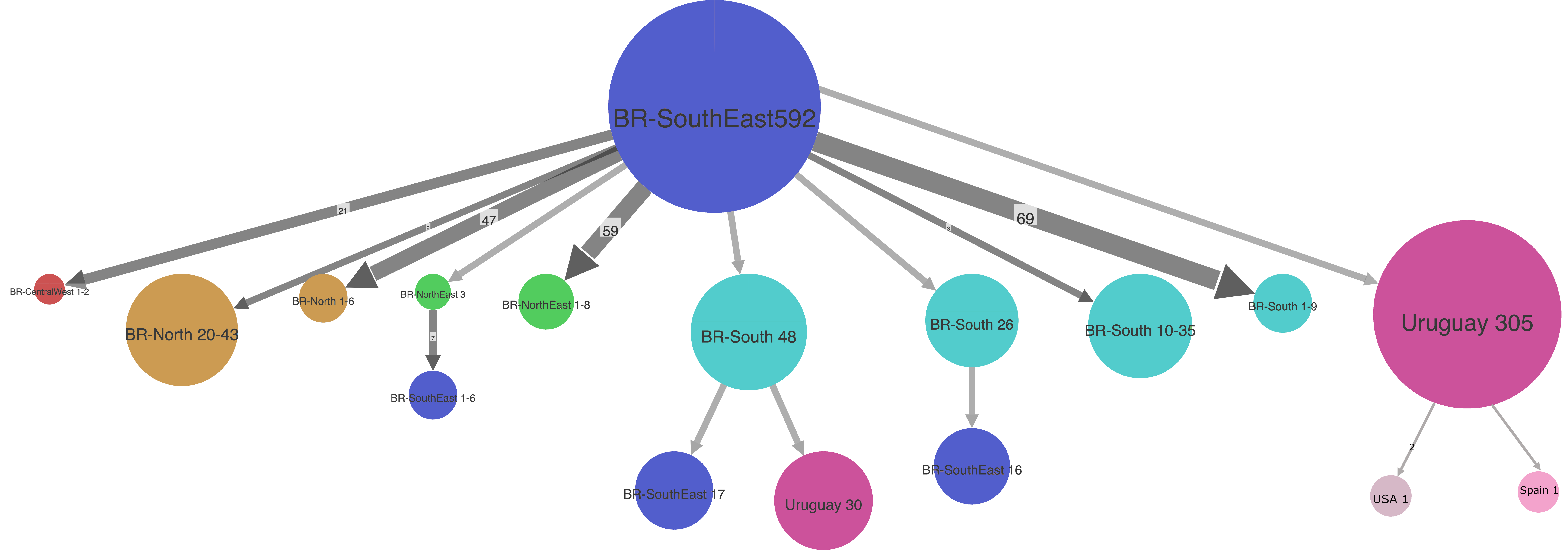
BR-SouthEast592
21
2
47
59
69
3
BR-CentralWest 1-2
BR-North 20-43
BR-North 1-6
BR-NorthEast 3
7
BR-SouthEast 1-6
BR-NorthEast 1-8
BR-South 48
BR-South 26
BR-South 10-35
BR-South 1-9
Uruguay 305
BR-SouthEast 17
Uruguay 30
BR-SouthEast 16
2
USA 1
Spain 1
